# Early Capture of Attention by Self-Face: Investigation Using a Temporal Order Judgment Task

**DOI:** 10.1177/20416695211032993

**Published:** 2021-07-29

**Authors:** Aditi Jublie, Devpriya Kumar

**Affiliations:** Department of Humanities & Social Sciences, Indian Institute of Technology Kanpur, Kanpur, India; Department of Cognitive Science, Indian Institute of Technology Kanpur, Kanpur, India

**Keywords:** attentional capture, face perception, self-face advantage, temporal order judgment

## Abstract

Earlier work on self-face processing has reported a bias in the processing of self-face result in faster response to self-face in comparison to other familiar and unfamiliar faces (termed as self-face advantage or SFA). Even though most studies agree that the SFA occurs due to an attentional bias, there is little agreement regarding the stage at which it occurs. While a large number of studies show self-face influencing processing later at disengagement stage, early event-related potential components show differential activity for the self-face suggesting that SFA occurs early. We address this contradiction using a cueless temporal order judgment task that allows us to investigate early perceptual processing, while bias due to top-down expectation is controlled. A greater shift in point of subjective simultaneity for self-face would indicate a greater processing advantage at early perceptual stage. With help of two experiments, we show an early perceptual advantage for self-face, compared to both a friend’s face and an unfamiliar face (Experiment 1). This advantage is present even when the effect of criterion shift is minimized (Experiment 2). Interestingly, the magnitude of advantage is similar for self-friend and self-unfamiliar pair. The evidence from the two experiments suggests early capture of attention as a likely reason for the SFA, which is present for the self-face but not for other familiar faces.

Self-faces are among the most salient stimuli that we come across and process frequently (Apps et al., 2015; Devue & Brédart, 2008). The self-face holds a special status and consistently shows a processing advantage compared to both an unfamiliar (Tong & Nakayama, 1999) and other familiar faces (Sui et al., 2015). Moreover, the ability to recognize self-face is considered as a sign of self-awareness in humans as well as nonhuman primates (Heschl & Burkart, 2006). A pertinent question that has engaged the attention of several researchers investigating self-face processing is the reason for this processing advantage (Ma & Han, 2010; Wang et al., 2011). Several factors have been pointed out in previous research, for instance, a better structural encoding of self-face (Keyes & Brady, 2010; Keyes et al., 2010; Qian et al., 2017), a greater familiarity with self-face (Bortolon et al., 2018; Sui et al., 2006), or a multimodal representation of self-face (Ambrosini et al., 2019; Apps et al., 2015; Northoff, 2011; Thirioux et al., 2016). Although many studies, including a recent meta-review (Bortolon & Raffard, 2018), observe a significant advantage in identifying self-face, the stage at which self-face affects perceptual processing is not clearly understood. Early behavioral investigations have found little evidence for automatic capture of attention by the self-face (Devue & Brédart, 2008; Keyes & Dlugokencka, 2014). Although recent electrophysiological studies suggest that the self-face might be able to both capture and retain attention at an early stage (Alzueta et al., 2020; Wójcik et al., 2018), behavioral support for capture of attention by self-face remains elusive. One possible reason for the mixed results might be the inability of previous paradigms to behaviorally dissociate between different stages of face processing. To address this issue, our article investigates the self-face’s ability to capture attention using a cueless temporal order judgment (TOJ) paradigm. In the standard TOJ task, participants are asked to specify which of the two sequentially presented stimuli, S1 and S2, separated by a variable stimulus onset asynchrony (SOA), occurred first. Using the TOJ paradigm, we can investigate how saliency of the stimuli influences perceptual processing when attention is equally distributed between multiple targets while controlling for other confounds. However, before we describe the current study, we first review previous work on attention and the self-face advantage (SFA).

Most of the studies on SFA and attention have focused mainly on tasks involving selective attention, wherein self-face is presented either as a target or a distractor, and the facilitation/interference caused by the presence of self-face is evaluated (Chakraborty & Chakrabarti, 2018; Devue & Brédart, 2008; Devue et al., 2009; Keyes & Dlugokencka, 2014; Wójcik et al., 2018). One of the first studies that show attentional capture due to self-face looked at the name–face interference when one’s own name is shown as the target flanked by a classmate’s face, compared to when a classmate’s name is shown as the target flanked by self-face (Brédart et al., 2006). Authors suggest that the self-face’s attention-grabbing properties might be due to a higher emotional value (Vico et al., 2010) or a greater familiarity (Bortolon et al., 2018) associated with one’s own face. Other studies fail to find sufficient evidence for attentional capture by the self-face in the condition of inattention (Laarni et al., 2000), or when the self-face is presented outside the focus of attention (Devue & Brédart, 2008). One possibility is that the self-face acts as a distractor, not because it captures attention but rather due to a greater difficulty in disengagement of attention. With the help of a visual search task, Devue et al. (2009) found that self-face influences the processing only when (a) participants look at it or (b) when self-face is the target. Further, this benefit is not limited to the self-face; other highly familiar faces also show a similar effect, putting uniqueness of self-face into question. On the other hand, results from electroencephalography studies suggest that the self-face differentially influences early visual processing. ERP components linked with early visual processing (N170, P2) and attention (N2PC, SPCN) show a modulation specific to self-face supporting the automatic capture of self-face (Alzueta et al., 2020; Keyes et al., 2010; Wójcik et al., 2018). Interestingly, these modulations are also present for faces for which have been synchronized with self-face to induce enfacement illusion (Estudillo et al., 2018). Furthermore, they do not find similar modulation for other familiar faces, hinting toward a unique ability of self-face to capture attention.

One possibility is that the discrepancy between studies that show an SFA, and those that fail to find support for SFA, might be due to inherent differences in the task which participants performed. In studies where the self-face processing was task-related, the SFA is either absent or appears at a later stage (Keyes & Dlugokencka, 2014), whereas studies where self-face processing was not task-relevant report an early effect of attention on SFA (Wójcik et al., 2018). Another factor that might influence SFA is the participant’s top-down expectation. When participants are asked to identify whether a photograph is self-related or not, a low-frequency alpha power (8–9 Hz) is observed even before the photograph is presented, suggesting that expecting a self-face biases changes brain activity even before the face is encountered. Further, the alpha band activity correlates with the participant’s judgment of self-relatedness (Lane et al., 2016). Another study (Alzueta et al., 2020) reports that by asking participants to perform an identification task researcher might predispose participants to detect the face as self-face, resulting in faster response times (RTs), a slower disengagement of attention from self-faces, and the SFA (Devue et al., 2009; Wójcik et al., 2018). One possible reason for this inconsistent finding might be that perceptual saliency operates at an early stage of processing while task expectation might operate at later stages.

We wanted to investigate the stage at which self-face influences early perceptual processing. By looking at the perceptual processing while controlling the task expectations, we might gain a greater insight into the different stage of self-face processing. Our aim was to directly test the idea that self-face can influence perceptual processing at an early stage. To do so, we use a cueless TOJ task (Constable et al., 2019; Donovan et al., 2011; Rajsic et al., 2017; Truong et al., 2017). Previously, cueless prior-entry effect has been used to look at preattentive processing of learned value (Rajsic et al., 2017), ownership (Constable et al., 2019; Truong et al., 2017), and fearful faces (West et al., 2009). In a TOJ paradigm, participants indicate (using a two-alternative forced choice task) which stimuli appeared first. Findings with exogenous cueing suggest the stimuli that receive attentional prioritization are perceived earlier (Shore et al., 2001). Although there is some debate regarding the mechanism responsible for the prior-entry effect, the majority view is that the prior-entry effect rises from early perceptual (Vibell et al., 2007) and attention-induced enhancements (Schneider & Bavelier, 2003; Spence & Parise, 2010) in sensory processing. The enhanced sensory processing is accompanied by an increase in the amplitude of early ERP components (P1, N1, P2), later represented as a difference in time perception. Using a cueless TOJ paradigm provides several advantages over other paradigms. First, it allows us to compare the processing of two stimuli when both the stimuli locations are attended simultaneously, hence providing a scenario where both the stimuli are processed and are equally relevant for the participant. Second, the time window of cueless TOJ allows investigating preattentive processing with little interference from later mechanism involving delayed disengagement. Third, as there are no cues to direct attention toward one of the two stimuli, the expectation bias cannot explain the perceptual advantage (Rajsic et al., 2017).

Experiment 1 investigates early capture of attention by the self-face, resulting in a prior-entry effect. To do so, we gave participants a cueless TOJ task for face pairs (with faces within the pair having asynchronous onset but synchronous offset) presented in random order and locations. These face pairs differed in the faces’ identity with all combinations of self-face, friend’s face, and a gender-matched unfamiliar face presented randomly in an equiprobable manner. Participants indicated whether the left or the right face appeared first. We measured the “prior-entry effect” by looking at individual and population-level logistic fits and used them to observe shifts in point of subjective simultaneity (PSS). A shift in PSS value toward the self-face would be indicative of a prior-entry of self-face, suggesting an early capture of attention by self-face.

Even though a shift in the PSS often indicates enhanced early sensory processing, decision processes such as a shift in criterion might also influence the results. Previous work on prior entry of self-owned objects suggests that this might be the case for self-related stimuli (Constable et al., 2019). When participants make TOJ between two objects, they report seeing self-owned objects earlier than an object they do not own. The authors observe this prior entry for a self-owned object when judgment is aligned with the measure of interest. Interestingly no prior-entry effect is observed when judgments are on an orthogonal dimension (when authors look at the preference for the self-owned object, and the question is about the position of the object perceived as arriving earlier). To minimize the effect of decision criterion in the observed PSS, we conducted a second experiment, in which the participants, similar to the first experiment, saw a face pair but gave a response on an orthogonal dimension (facial expression) to the identity of face (Bate & Bennetts, 2015; Kumar & Srinivasan, 2011).

## Experiment 1

As discussed earlier, the experiment’s goal was to investigate whether self-faces capture attention early. Participants indicated which face came first from a pair of asynchronously presented faces while we manipulated the SOA between faces (0 to 166 ms), the order of presentation, and the face pair (we used the self-unfamiliar face pair [SU], the self-friend face pair [SF], and the friend-unfamiliar pair [FU]). We randomized both the location and identity of the faces within a face pair. Randomization ensured that participants were equally likely to see self-face, friend’s face, or the unfamiliar face at either of the two possible locations, minimizing the effects of expectation. If the self-face captures attention early, we expect a prior-entry of self-face compared to friend’s face and the unfamiliar face, resulting in a shift in the PSS in favor of self-face. If this attentional capture is unique to self-faces, we should not observe a similar shift for FU face pair.

### Method

#### Participants

We planned the experiment to obtain a moderate effect size of 0.5 (Cohen’s *d*) and a power (1–beta) of 0.8 and alpha =.05. As we were interested in the shift in the PSS for self-face compared to other faces, we calculated power for a one-sample two-tailed *t* test. The sample size was calculated in “*R v3.5.3*” using function *pwr.t.test*() in the package “*pwr*” . The experiment was conducted on 36 participants (19 females, 17 males). All the participants were students of Indian Institute of Technology Kanpur (IITK) within the age range of 18–28 years (mean age= 23.5, *SD*= 3.26) with normal or corrected-to-normal vision. At the beginning of the study, all participants provided informed consent for the experiment and using their picture in the experiment. Participants were compensated with Rs.50 for the first phase (where we photographed them) and Rs.50 for participating in the main experiment. We conducted the study after approval from the Institute’s Ethics Committee of IIT Kanpur (IEC Communication No: IITK/IEC/2018-19/I/11).

#### Apparatus

The experiment was conducted on a standard IBM PC at a refresh rate of 60 Hz and a resolution of 1,024 × 768 on 24" LED display. Participants were seated at a distance of 60 cm from the monitor screen and gave their responses through a standard QWERTY keyboard. The experiment was designed using PsychoPy.

#### Stimuli and Procedure

Stimuli consisted of the grayscale face of participants (S), their friend (F), and an unfamiliar face (U), all having neutral facial expressions. Photographs with neutral and happy facial expressions of both the participant and their friend were taken before the experiment (see Supplemental material for details). In addition, we took photographs of two volunteers (one male and one female) from outside the institute; these were used as the unfamiliar face. All the images were cropped to an oval frame, edited to remove information other than the face, and matched for lower-level perceptual features such as contrast. These photographs were then rated by an independent set of observers on valence, intensity, arousal, and genuineness (see Supplemental material for details). For the first experiment, we used photographs with neutral facial expression. Faces were presented in the periphery at an angle 4° × 4° at an eccentricity of 6° from the center.

**Figure 1.**
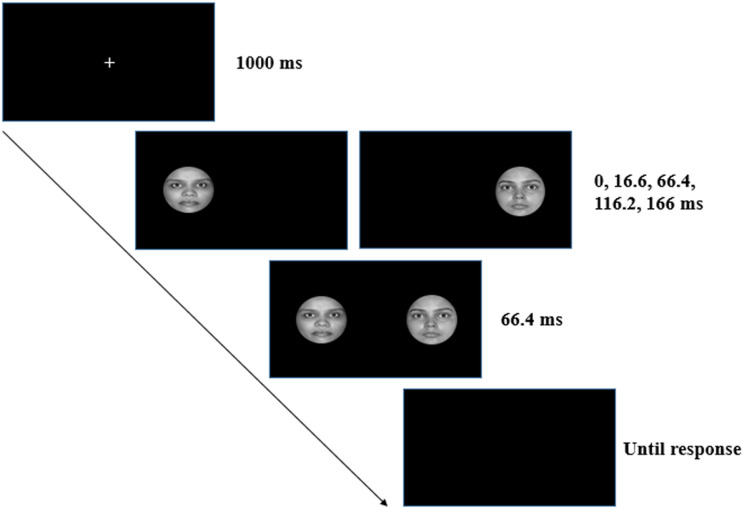
Trial structure. Participants were presented with a fixation cross followed by two faces presented with an asynchronous onset. The offset of both the faces was synchronized. Participants had to report which face appeared first: the left face or the right face.

The experiment was conducted in two phases. In the first phase, participants were informed about the nature of the study, and after obtaining consent, pictures of participant and their friend were taken under controlled condition (see Supplemental material for details). The experiment took place in the second phase; in most cases, both participant and their friend took part in the experiment. During the second phase, participants were instructed that they will see two faces on screen, and these can be either their face or their friends face or an unfamiliar face. They were asked to report whether the “left face” or the “right face” appeared first by pressing z and m key, respectively. Each trial began with presentation of a fixation cross that lasted for 1,000 ms. This was followed by the presentation of the first face either on the left or on the right side of the fixation cross. After a variable interval of 0, 16.6, 66.4, 116.2, and 166 ms, the second face was presented. The two faces belonged to the self-unfamiliar pair, the self-friend pair, or the friend-unfamiliar pair. Each pair was presented equal number of times in random order. The face pair remained on screen for 66.4 ms (four frames after the onset of the second image) and followed by a blank screen where the participant had to indicate as quickly as possible whether the left or the right face appeared first by pressing the “z” and “m” key, respectively. The blank screen remained until a response was made (see Figure 1 for the trial structure). The order of presentation and location was counterbalanced across trials. Each participant completed a practice block of 60 trials, followed by the main experiment having 720 trials (5 SOAs × 2 Location Order × 3 Face Pairs × 24 Repetitions).

The main experiment was divided into four blocks with a compulsory break in between the blocks. For each trial, we recorded the accuracy and RT of response. The total experiment took around 30 minutes to complete.

### Results and Discussion

#### Data Analysis

For each participant, responses beyond 1,000 ms participants’ mean RT were considered outliers and removed from further analysis; this resulted in a loss of approximately 1% of trials from the overall collected data. On average, at the longest SOA of 166 ms, the participants achieved an accuracy of 93.2%, 93.1%, and 93.9% for the FU, SF, and SU face pairs, respectively. The remaining data were recoded to calculate the proportion of trial participants reported seeing the preferred face first (when participants reported the order as SU in SU and US, SF in SF and FS, and FU in FU and UF). The preferred face in each face pair was the face which was of interest to us, and the preference for the face would be indicated by a negative shift in PSS.

We fit a logistic curve of the form “*y = 1/exp(–β × (x–α)))*” to the participant’s data looking at proportion of trials in which participants perceived preferred face earlier as a function of SOA between the two faces. The parameters α and β refer to the threshold and slope of the logistic function.

For curve fitting, we used “*quickpsy()*” function in R, which estimates the best fit using the maximum likelihood estimation method. To assess the goodness of fit, we calculated the deviance for all 108 curves (36 participants × 3 face pairs). The deviance was not significant (*p* > .01, the *p* was calculated using bootstrap across 2,000 samples) for any of the fits (Linares et al., 2018). The PSS was the SOA for which participants reported seeing each of the face pair with an equal likelihood (Spence & Parise, 2010).

#### Shifts in PSS

**Figure 2.**
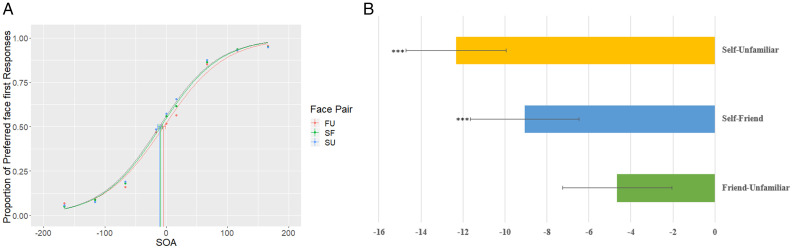
A: Population fit for Experiment 1. For each participant, we calculated the proportion of trials in which participants report seeing preferred face first. Data from all the participants were cleaned and pooled to obtain population-level PSS estimates for the three face pairs. The figure suggests that while participants report seeing self-face earlier than an unfamiliar face and friend’s face, this is indicated by a negative PSS value for SU and SF conditions. A similar shift in the PSS is not present for FU face pair. B: Average PSS values for each face pair in Experiment 1.***indicates significance at p = 0.001. SOA = stimulus onset asynchrony; FU = friend-unfamiliar pair; SF = self-friend face pair; SU = self-unfamiliar face pair.

We conducted a one-way repeated measures analysis of variance for shift in PSS across the three levels. We did not find a significant difference between face pairs, *F*(2, 70) = 2.27, *p* = .11, *µ_p_*^2^ = .061. To determine if there were any statistically significant shifts for any of the face pairs, we conducted a one-sample *t* test for the pairs SU, SF, and FU. We found a significant shift for the SU pair—mean shift = –12.32 ms, *t*(35) = –5.186, *p*=.001, 95%, CI [–17.14 ms, –7.49 ms], *d =* 0.86—and SF pair—mean shift = –9.06 ms, *t*(35) = –3.522, *p* =.001, 95% CI [–13.04 ms, –2.49 ms], *d* = 0.58 (see Figure 2A and B). Participants perceive their own face as appearing earlier than a friend’s face (9 ms) and an unfamiliar face (12 ms). For FU face pair, this shift was nonsignificant, mean shift = –4.65 ms, *t*(35)= –1.16, *p*=.11, *d* = 0.26.

**Figure 3.**
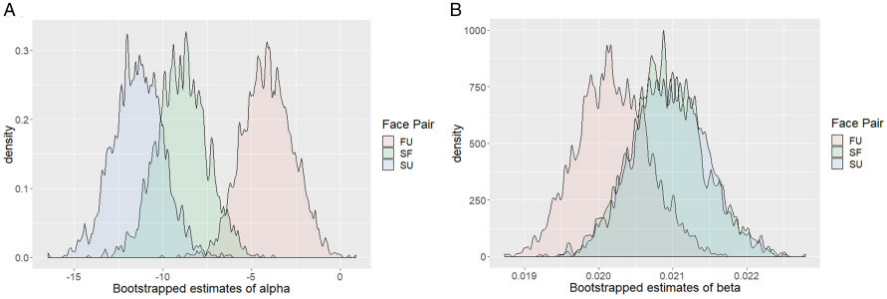
Bootstrap estimates of parameters of the psychophysical function. To get better estimates of the parameters, we bootstrapped the data across 2,000 samples. We find that (A) the parameter alpha (estimate of the threshold) is significantly lower for SO and SF face pairs compared to FO face pair and (B) the smaller estimate of parameter beta (measure of slope) suggests that discrimination is difficult between friend’s face and the unfamiliar face. However, the difference between slope estimates of the three face pair is not significant. FU = friend-unfamiliar pair; SF = self-friend face pair; SU = self-unfamiliar face pair.

#### Population Analysis

To further investigate the shifts in PSS observed in individual data, we used bootstrap estimates for the three face pairs assuming all data from a single observer (bootstrapped across 2,000 samples). Looking at population-level analysis has advantages over the two step-method used classically (that involves calculating individual thresholds and then analyze variance in threshold across participants). Population-level analysis allows comparison of within subject conditions, has a greater power, and allows easy calculation and interpretation of population confidence intervals (Moscatelli et al., 2012). The population analysis uses bootstrapping to calculate the estimates of parameters alpha and beta, along with a 95% confidence interval for these estimates. A negative shift in parameter alpha for SF and SU face pairs compared to FU face pair (nonoverlapping confidence for FU compared to SF and SU) would indicate a processing advantage for self-face compared to friend’s face and an unfamiliar face.

The data show that an SFA is present against both friends face—mean shift= –10.56, 95% CI [–12.9, –7.6]—and an unfamiliar face—mean shift = –13.44, 95% CI [–16.68, –10]. A small difference is observed for the friend’s face when paired with the unfamiliar face—mean shift= –4.59, 95% CI [–7.5, –1.9]—however, this difference was less than the shift in PSE observed for SU and SF conditions (see Figure 3A and B). The mean shift estimates for population-level analysis are close to the threshold averages obtained from individual fits. The results suggest that participants report perceiving their face nearly 13 ms earlier than an unfamiliar face and 10 ms earlier than a friend’s face. Even though small, the advantage for FU pair and the smaller advantage for SF face pair as compared to SU face pair, nonetheless hints toward the possibility that familiarity with the face might contribute to this processing advantage. The self-reference advantage does not seem to be unique for self-face.

Results show a prior-entry effect for self-face, that is, there is a processing advantage for self-face compared to both a friend’s face and an unfamiliar face, supporting our hypothesis that self-face captures attention early. Although a prior entry is also observed for friends’ face, the effect seems to be relatively small. The results clearly show that even when no prior expectation is present, and participants need to process both the faces for a successful response, we see an SFA. These results support our hypothesis and show a prior-entry effect for self-faces in a cueless TOJ task. These findings provide clear evidence that self-face not only influences processing when participants are paying attention to them but can also capture attention and influence processing at an early stage. These results are in line with recent studies suggesting that self-face affects perceptual processing at an early stage. Although before we can conclusively say that SFA observed in Experiment 1 provides evidence that the self-face captures attention early, we must rule out other possible explanation of criterion shift for observing this effect.

## Experiment 2

Recent work on the prior-entry effect suggests that although attentional prioritization does play a significant role in the prior-entry effect observed using TOJ paradigm, it might not be the only factor that influence participants’ response. The shift in PSS might also be a result of a shift in decision criterion due to an active processing of self-relevant stimuli (Constable et al., 2019). To minimize the effect of criterion shift on prior-entry, earlier studies have employed orthogonal TOJ task where the dimension of response does not require active processing of self-relevant stimuli (Constable et al., 2019). When orthogonal TOJ is used, the prior-entry effect, at least for self-owned object, disappears, suggesting that criterion shift might also explain why we observe a shift in PSS for self-faces. The task given to participant requires them to actively process the identity of the face, which would suggest that the SFA might be due to a top-down expectation, rather than early perceptual processing.

The aim of Experiment 2 was to investigate prior-entry effect for self-face when role of criterion shift is minimized. To do so, we gave participants an orthogonal TOJ task where participants were asked to respond on the basis of facial expression of the face. Bate and Bennetts (2015) have shown that emotional expression and face identification are dissociable; therefore, by using an emotion judgment task that is orthogonal to the domain of interest, that is, face, we can ensure that the bias received for the domain of interest is not intentional in nature.^1^ In Experiment 2, instead of asking participants to report which face appeared first, we asked participants to report the facial expression of the face that appeared first. As we observed nonsignificant prior-entry effect for friend’s face (compared to unfamiliar face) in previous experiment, to simplify the design, we presented only the SU and SF face pairs. These face pairs not only differed in identity of the face but also the expression of each face (happy and neutral) within the face pair. We hypothesized that if the prior-entry effect observed in Experiment 1 indicates a perceptual processing advantage, the PSS shift for self-face should be present even when participants perform the orthogonal task.

### Method

#### Participants

We planned the experiment for an effect size (Cohen’s *d*) of 0.5, a power (1–β) = 0.8, and a significance level (α = .05). Sample size was calculated for a one-sample two-tailed *t* test in *R* using the function “*pwr.t.test()*” from package “*pwr*.” Sample size thus obtained was 33. The experiment was conducted on 33 students (16 females, 17 males) of IITK, within the age range of 18–28 (mean= 24.6, *SD* = 3.32) and with normal or corrected-to-normal vision. Twenty-seven of these participants had also taken part in Experiment 1. To minimize any influence of Experiment 1 on results of Experiment 2, the two experiments performed after a gap of 2 months. Participants were compensated (Rs.50) for participation in the experiment.

#### Stimuli and Apparatus

Stimuli were similar to those used in Experiment 1, with the only difference being that in addition to neutral faces, we also included faces with happy facial expression. The apparatus’ and other details were the same as Experiment 1.

#### Procedure

Participants performed the orthogonal response paradigm task where each trial began with a blank screen (for 1,000 ms to minimize after image effect) followed by a fixation cross that lasted for 1,000 ms, after which a face was presented either on the left or on the right side of the fixation cross, and after a variable interval of 0 ms, 16.6 ms, 66.4 ms, 116.2 ms, and 166 ms, the other stimulus was presented. Both the images remained on screen for 66.4 ms (four frames after the onset of the second image). This was followed by a blank screen where the participants had to respond as quickly as possible. The instructions and procedure were similar to the first experiment. The only difference was in the task participants were asked to perform. Participants were told that they will be presented with a face pair and had to indicate the expression of the face that appeared first, by pressing the z key if the happy face appeared first, and m key if the neutral face appeared first.

The face pair was either the self-happy versus friend-neutral pair (SH-FN), self-neutral versus friend-happy pair (SN-FH), self-happy versus unfamiliar-neutral pair (SH-UN), or self-neutral versus unfamiliar-happy (SN-UH). Each pair was presented equal number of times in random order. The order of presentation of face pairs in left and right locations were randomized across trials. Each participant completed a practice block of 15 trials, followed by the main experiment having 432 trials. Each face pair had 216 trials. The main experiment was divided into three blocks, with compulsory breaks in between.

### Results and Discussion

Data cleaning and fitting criteria used were same as those in Experiment 1. Fit for data from three participants failed to converge and was not considered for further analysis. To assess the goodness of fit, we calculated the deviance for all 90 curves (30 participants × 3 face pairs). The deviance was not significant (*p* > .01, the *p* was calculated using bootstrap across 2,000 samples) for any of the fits (Linares et al., 2018). We estimated the PSS, which was the SOA for which participants reported seeing each of the face pair with equal likelihood (Spence & Parise, 2010).

#### Shift in PSS

**Figure 4.**
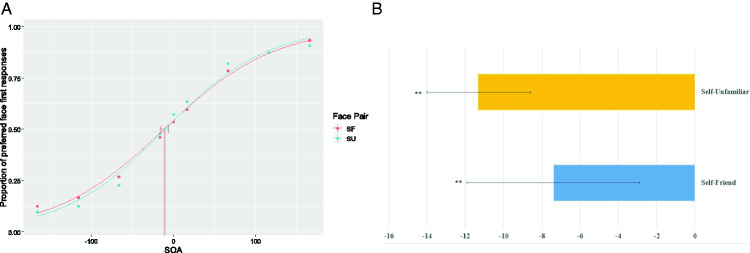
A: Population fit for Experiment 2. For each participant, we calculated the proportion of trials in which participants report seeing preferred face first. Data from all the participants were cleaned and pooled to obtain population-level PSS estimates for the two face pairs. The figure suggests that participants report seeing self-face earlier than an unfamiliar face and friend’s face, this is indicated by a negative PSS value for SU and SF conditions. B: PSS values for each face pair in Experiment 2.**indicates significance at p = 0.01. SOA = stimulus onset asynchrony; SF = self-friend face pair; SU = self-unfamiliar face pair.

We found a significant shift in PSS (from 0) for the pairs self-unfamiliar, mean shift = –7.39 ms, *t*(29) = –2.75, *p* = .01, 95% CI [–12.89 ms, –1.9 ms], *d* = 0.51 (see Figure 4B) and nonsignificant shift for self-friend face pair, mean shift = –11.31 ms, *t*(29) = –2.75, *p* = .01, 95% CI [–19.72 ms, –2.89 ms], *d =* 0.51. Results indicate that although the PSS shift is observed even with the orthogonal task, the effect size is reduced. Although in both the experiments we obtained a large effect for SU face pair (the effects size for SU face pair reduced from 0.86 in Experiment 1 to 0.51 in Experiment 2), the effect size for SF pair decreased from a 0.58 in Experiment 1 to of 0.51. The huge reduction in effect size for SU pair but not for the SF pair suggests that the difference between SF and SU pair observed in the first experiment might be due to familiarity having an effect at later stages of processing in form of response bias.

#### Population Analysis

**Figure 5.**
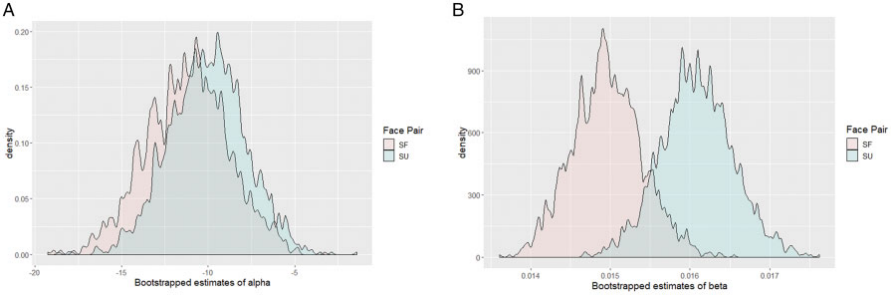
Bootstrap estimates of parameters of the psychophysical function. To get better estimates of the parameters, we bootstrapped the data across 2,000 samples. We find that (A) the parameter alpha (estimate of the threshold) is similar for SO and SF face pairs, suggesting a comparable threshold, and (B) the smaller estimate of parameter beta (measure of slope) suggests that discrimination is difficult for SF face pair compared to SU face pair. However, the difference between slope estimates is not significant. SF = self-friend face pair; SU = self-unfamiliar face pair.

We next estimated the confidence interval for the threshold at the population level by bootstrapping data across 2,000 samples. The estimates of the population mean from bootstrap analysis are close to the means obtained from individual fits. There was a mean shift was –10.01 (95% CI [–14.41, –5.79]) for self-unfamiliar face pair and –11.27 ms (95% CI [–15.41, –5.78]) for self-friend face pair. Results suggest an identical advantage for the self-face in comparison to both friend’s face and the unfamiliar face (see Figure 5A and B).

Earlier we hypothesized that if the advantage observed in Experiment 1 is due to bias at early perceptual level, then we should see a prior entry for the self-face even when participants are responding on orthogonal dimension. Results support our hypothesis, supporting the idea that there is an early perceptual bias for self-face. Nevertheless, it is important to note that there is a decrease in effect size of SFA from Experiment 1 to Experiment 2.

## General Discussion

The role self-relatedness plays in influencing the early stages of processing is not clear. While some studies have suggested an early automatic capture of attention (Bola et al., 2021), others have either failed to find SFA at the early perceptual level or report that advantage occurs only under certain contexts (Devue et al., 2009). Given the inconsistency in findings related to the SFA, the current study provides a firm footing to the idea that not only does SFA exist, but it also occurs at an early level. Our results support an automatic, unintentional capture of attention by self-face. These findings are in line with recent electrophysiological studies that report early ERP components modulated by self-face (Bola et al., 2021; Wójcik et al., 2018).

We used a cueless TOJ task to investigate perceptual processing of self-face. With the help of two experiments, we see that the participants report perceiving self-face earlier compared to a friend’s face or a stranger’s face. We find this advantage both for normal as well as orthogonal TOJ, suggesting that self-face captures attention early. However, the decrease in the effect size for second experiment suggests that early capture of attention might not be the only factor determining SFA. What can we learn about the processing of self-face from these results? Why does self-face show prior-entry?

Attention is thought to influence classical TOJ in three ways: (a) by interaction of cue and stimuli at the attended location, (b) by reducing transmission time of the attended location, and (c) by affecting decision mechanism (Schneider & Bavelier, 2003). Two types of models have been used to explain these effects of attention on TOJ. First are the arrival time-based models that explain TOJ as emerging from competition between two independent signals (in our case, the two faces in the face pair), comparing the relative arrival latencies at the decision-making stage (Sternberg & Knoll, 1973). Shift in PSS in such case would occur at the decision-making stage due to transmission speed of the signal. However, if this was the case, minimizing of criterion shift (Experiment 2) should not have influenced the shift magnitude. More recent models focus on relative timing judgments (Yarrow et al., 2011), suggesting that it is difficult to imagine how neural transmission speed can be significantly affected by attention in a relatively hardwired system. A better explanation of TOJ is through a race-based model of attention for evidence accumulation where race operates on shorter time-scales equivalent to those of the SOA manipulations being used in the TOJ task (Aghdaee et al., 2014). In such a model, a bias in TOJ due to attention can result from (a) better sensory integration of information rather than increase in propagation speed (resulting in faster accumulation of information for one of the stimuli), or (b) by lowering the threshold for detection of one of the stimuli (resulting in earlier detection), or (c) by giving a starting advantage to one of the stimuli (by cueing a particular location). Although the model just described is largely based on data from study of receptive field properties of neurons in monkeys, it has important implications for our study. But before we explain the link, let us eliminate other possible explanations of the observed effect.

Our results cannot be explained by using a cue-stimulus interaction approach as we use a cueless TOJ task. Hence, the cause of the observed shift in PSS should lie in the intrinsic property of the stimuli, even though the question about what intrinsic property of the self-face causes this shift still remains. We can also safely eliminate decision-making mechanism as a possible explanation. Unlike other studies on self-owned objects (Constable et al., 2019), we continue to observe a shift in PSS for SFA (although reduced) even when effect of criterion shift is minimized (Experiment 2).The only possible mechanism by which we can explain our results is through increase in propagation time. As discussed earlier, from a neural point of view, it does not make sense to assume that attention can influence the speed at which signal propagates through a relatively hardwired system. However, it is still possible to improve the overall transmission duration by having a greater integration of sensory evidence for the self-face, given that participants have to take decisions on short time-scale and for stimuli differing in onset in the order of tens of milliseconds. The face that accumulates sensory evidence faster would win the short race (the race would be influenced more by early perceptual processes compared to later stages). This of course does not eliminate the possibility that later stages may also influence the final decision (as evidenced by a greater effect size in Experiment 1 when effect of criterion shift was not minimized.)

We speculate that the difference in initial sensory representation of self-face might be the intrinsic property of the self-face that enhances the transmission time of the information, resulting in prior entry of self-face. Previous research supports our speculation. The self-face recognition is better when accompanied by interoceptive heartbeat signal (Ambrosini et al., 2019). Activation sensory and motor association cortices have been reported for tasks involving self-face/self-body recognition (Sugiura et al., 2006). It has been argued that the self-face processing depends on multisensory processing of information, combining the visual, proprioceptive, motor, and tactile information (Sugiura, 2013), and these multisensory interactions are known to effectively modulate attentional capture (Tang et al., 2016). Dual representation of self-face might be another reason for the enhanced processing. Self-face representation is found to be dependent on both featural as well as holistic processing (Hills, 2018; Keyes, 2012). Participants show greater number of fixations on self-face compared to familiar faces, indicating a greater featural processing for self-face (Hills, 2018). More evidence for this idea comes from the observed lack of face inversion effect for self-faces (Yin et al., 2018), suggesting featural processing as one of the possible reasons for SFA. Interestingly, they report a decrease in SFA for inverted faces in adults (even though the advantage is intact for upright faces), suggesting role of both holistic and featural processing in SFA. Based on current results, we cannot comment on the exact mechanism underlying SFA. However, future studies can be conducted to have a greater understanding of how self-face is represented and how this may result in early processing advantage for self-faces.

To summarize, we show clear evidence that the self-face influences perceptual processing at an early stage, and the use of TOJ task allows us to investigate this early processing in isolation. This advantage for self-face is not because of expectation bias or the nature of the task, as we observe this benefit even when participants’ judgment does not depend on the face’s identity. An important consideration is the uniqueness of the self-face. Admittedly, the familiarity as a confound in self-face processing has long been a significant confound in all self-face studies. This study, although hinting toward the idea that effects of familiarity influence processing later, still does not allow us to make strong claims about familiarity as not being a confound in self-face processing. Nevertheless, future studies using the cueless TOJ paradigm can investigate the possible manipulations that would allow us to tease apart effects of familiarity from those of self-face.

## Supplemental Material

sj-pdf-1-ipe-10.1177_20416695211032993 - Supplemental material for Early Capture of Attention by Self-Face: Investigation Using a Temporal Order Judgment Task Click here for additional data file. Supplemental material, sj-pdf-1-ipe-10.1177_20416695211032993 for Early Capture of Attention by Self-Face: Investigation Using a Temporal Order Judgment Task by Aditi Jublie and Devpriya Kumar in i-Perception

## Note

1 To verify that emotion detection is orthogonal to face identification in our paradigm, we performed an experiment where participant was asked to report order of the faces (which face came first) while we manipulated facial expression (Happy or Neutral) and identity (Self-face and Unfamiliar face). Results suggest that the shift in PSE due to SFA is not influenced by emotional valence of the face.
