# Supplementary material for: Early Capture of Attention by Self-Face: Investigation Using a Temporal Order Judgment Task
Source: Iperception. 2021 Jul 29;12(4):20416695211032993. doi: 10.1177/20416695211032993 (PMC8327255; doi:10.1177/20416695211032993)
Supplement: sj-pdf-1-ipe-10.1177_20416695211032993 - Supplemental material for Early Capture of Attention by Self-Face: Investigation Using a Temporal Order Judgment Task [file sj-pdf-1-ipe-10.1177_20416695211032993.pdf]

Supplementary Information

Details of Stimuli used in study

The nature of the study was explained to the participants before phase 1. In phase 1, participants were requested to get their friends along with them. Consent for taking photographs was obtained from both the participants and their friends, along with the consent to take part in the experiment. In most cases, both the participant and their friend took part in the main experiment. A professional photographer took pictures (exhibiting happy, sad, or neutral expression) in controlled settings at Media Lab, IIT Kanpur. A total of 55 participants were photographed. These photographs were cropped to an oval frame to remove facial hair and any other identifications converted to grayscale and matched for contrast using a custom script designed in MATLAB (Kumar & Srinivasan, 2011). Fifteen other participants (who did not take part in the experiment and were not photographed) categorized the facial expression as' sad',' happy', 'neutral', and rated these photographs on a five-point rating scale for valence intensity, arousal, and genuineness and emotion category. The photographs were presented in randomized order, and each participant rated each picture five times for every domain.We did not find any difference in the rating between self-faces, friends face, unfamiliar face on any of the rating parameters.
